# Neurorehabilitation Across Continents: the WFNR-EFNR Regional Meeting in conjunction with the 19^th^ Congress of the Society for the Study of Neuroprotection and Neuroplasticity and the 19^th^ International Summer School of Neurology in Baku, Azerbaijan

**DOI:** 10.25122/jml-2024-1014

**Published:** 2024-09

**Authors:** Stefana-Andrada Dobran, Alexandra Gherman

**Affiliations:** 1 RoNeuro Institute for Neurological Research and Diagnostic, Cluj-Napoca, Romania; 2 Department of Neuroscience, Iuliu Hatieganu University of Medicine and Pharmacy, Cluj-Napoca, Romania

## Introduction

This summer, the vibrant capital of Azerbaijan, Baku, hosted three prestigious neuroscience events, from July 10^th^ to 11^th^, the WFNR-EFNR Regional Meeting in conjunction with the 19^th^ Congress of the Society for the Study of Neuroprotection and Neuroplasticity (SSNN) and the 19^th^ International Summer School of Neurology. These converged to offer international perspectives and provide a platform for exchanging knowledge, sharing research findings, and innovating neurorehabilitation and brain recovery practices.

The comprehensive agenda focused on fundamental principles of neuroprotection and neurorehabilitation, along with practical applications, highlighting the biological and psychosocial impact of common neurological disorders and injuries such as stroke and traumatic brain injury (TBI). Moreover, it explored the complexities of rare neurological disorders and discussed emerging technologies and strategies for neurorehabilitation that are increasingly implemented on larger scales. A course on vestibular rehabilitation provided practical insights for managing balance disorders and improving patient outcomes.

A central focus in neurorehabilitation, stroke has become a critical issue in neurology due to its rising prevalence worldwide, complexity, and impact on health and quality of life. Stroke affects numerous neurological functions and leads to a range of consequences, such as dysphagia, aphasia, pain, fatigue, spasticity, and cognitive and neuropsychological issues [1-4].

## Advancements in neurorehabilitation: Insights from the WFNR-EFNR Regional Meeting together with the 19^th^ SSNN Congress and 19^th^ International Summer School of Neurology

**Figure 1:**
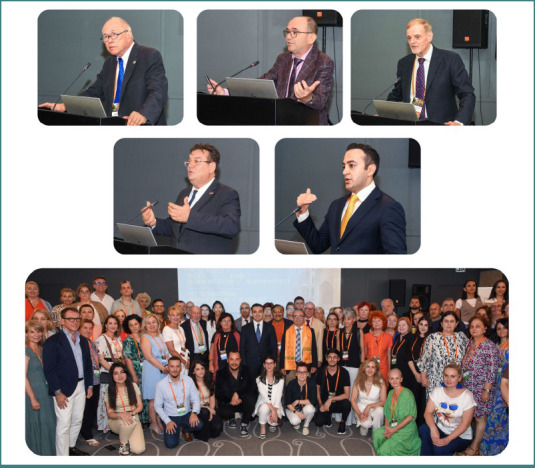
Photos from the WFNR-EFNR Regional Meeting in conjunction with the 19^th^ Congress of the Society for the Study of Neuroprotection and Neuroplasticity (SSNN) and the 19^th^ International Summer School of Neurology. First row - from left to right: Prof. Volker Hömberg, WFNR President, Prof. Dafin Mureșanu, EFNR President, Prof. Natan Bornstein, president of the Society for the Study of Neuroprotection and Neuroplasticity (SSNN) Scientific Committee; second row: HE Vasile Soare, Ambassador of Romania to the Republic of Azerbaijan, and Dr. Parvin Akbarov, Advisor to the chairman and administration of TABIB; bottom row: group photo.

The event was coordinated by Professor Dafin F. Mureșanu, the president of the European Federation for Neurorehabilitation Societies (EFNR), Professor Volker Hömberg, President of the World Federation for Neurorehabilitation (WFNR), and Professor Natan Bornstein, president of the Scientific Committee of the Society for the Study of Neuroprotection and Neuroplasticity (SSNN) (Figure 1). Speakers from Turkey, Romania, Italy, Germany, Poland, Hungary, India, Israel, Lebanon, and the United Arab Emirates together with over 250 participants from Europe and Asia attended the meeting (Annex A).

Annex A

The presidential session, a central part of the event, addressed innovative concepts and practices in neurorehabilitation, including future designs, robotics, neurotrophic factors, and brain reserve, with special attention to stroke and its consequences. The following lectures focused on key aspects of prevalent neurological disorders, including multiple sclerosis, TBI, movement disorders, and stroke, exploring recovery factors and the long-term effects of these conditions. Additionally, presentations explored pharmacological options, neuromodulation approaches to language investigation, the application of neurotechnology, and advancements in teleneurorehabilitation.

A teaching course on vestibular rehabilitation united experts to discuss the anatomy of balance, foundational principles, the management of patients with vertigo, and comprehensive assessment and treatment strategies. It is noteworthy that all lecturers engaged the audience in dynamic exercises and provided examples of clinical evaluation.

The EFNR-WFNR Round Table addressed current strategies in neurorehabilitation, through the lens of national and regional healthcare systems, and as specialized assessments from public health and clinical perspectives, with a focus on the results of clinical trials and insights into future guidelines and health strategies.

The final day concentrated on rare neurological diseases, covering essential information for general practitioners, addressing problems in diagnosis and therapy, and underlining the role of the World Federation of Neurology (WFN). Additionally, presentations explored teleneurorehailitation, therapy of mitochondrial diseases, and the impact of digital technologies and artificial intelligence (AI).

The Academy for Multidisciplinary Neurotraumatology (AMN), the RoNeuro Institute for Research and Diagnostic, and the Administration of the Regional Medical Divisions in Azerbaijan (TABIB) supported the event.

Experts in neurosciences, neurology, and neurorehabilitation from clinical, research, and educational backgrounds actively contributed to the scientific discourse. The event also aimed at expanding the WFNR and EFNR networks, as a prerequisite for improving regional neurorehabilitation, from fundamental research to innovative clinical practices.

“On July 10 and 11 of this year, the WFNR, together with EFNR, SSNN, and AMN, hosted an intriguing regional meeting in conjunction with our International Summer School of Neurology. We welcomed over 250 attendants from 11 countries and the program covered a broad range of pressing topics, from neuroplasticity research to neurology and neurorehabilitation. We were pleased with the wonderful hospitality of our hosts in Azerbaijan, and the meeting offered a chance to strengthen our relationship with our friends in central Asia. The high scientific quality of the program has made a lasting impact on the activities of all contributing societies, reinforcing our mission to improve education, research, and quality of services. As a new feature, we had a sparkling course on vestibular rehabilitation – a field sometimes overlooked in neurology. Also, a session on rare neurological diseases, another frequently neglected field, showed interesting and stimulating insights, including on Artificial Intelligence (AI) and telemedicine applications. The academic program was enriched with discussions with colleagues from scientific, clinical, and political backgrounds. Not least, we had a chance to explore the city of Baku and get a glimpse of a place with a long history of linking the East and West. I would like to extend my gratitude to the organizers, speakers, and participants for a brilliant meeting!”

*Prof. Dr. med.Dr.h.c. Volker Hömberg, WFNR President*

## WFNR-EFNR: Improving clinical practices, research, and education in neurorehabilitation

The WFNR and EFNR play a central role in improving neurorehabilitation practices and promoting education and research at the European and global levels.

The World Federation for Neurorehabilitation is a multidisciplinary organization that aims to improve awareness of neurorehabilitation, provide training and education, and encourage research and collaboration on a global level. The federation provides a forum for all professionals worldwide interested in neurorehabilitation, bringing together over 5,000 members, over 40 Special Interest Groups (SIGs), and partnerships with 41 national societies.

The European Federation of Neurorehabilitation Societies represents a forum for creating a scientific, academic, and work environment focused on patient care open to professionals interested in neurorehabilitation across Europe. It builds upon three pillars to create a multidisciplinary hub for knowledge exchange: (1) sustain and deepen the development of neurorehabilitation in all European countries and the interaction among stakeholders and decision-makers, (2) host a forum for all health professionals involved in neurorehabilitation across Europe, and (3) enable young clinicians to work on a joint platform and provide tools for education and training.

“In the context of the global increasing need for neurorehabilitation, an essential step would be the creation of new multidisciplinary national societies as key interfaces between global players and national healthcare providers. This encompasses efforts from academic bodies, healthcare practitioners, and public health specialists. The success of this complex endeavour lies in the perfect blend between human knowledge and artificial intelligence, converging for the well-being of our patients.”

*Professor Dr. Dafin. F. Mureșanu, EFNR President*

The close relationship between WFNR and EFNR is based upon common goals of enhancing awareness, providing training and education with the support of high-experience specialists, and fostering research and collaboration. The two Federations aim to join highly experienced professionals and young clinicians in the field of neurorehabilitation, which is reflected through the young European Federation for Neurorehabilitation (yEFNR). The SIGs within the WFNR – including yEFNR – focus on advancing specific areas of interest aligned with the Federation’s goals. The yEFNR is dedicated to connecting professionals in their early careers to promote research and education. Moreover, a mentorship program is designed to facilitate the transfer of knowledge and skills from the senior members to the new generation. The two entities organize or endorse congresses, teaching and training courses, and summer schools on various topics (e.g., stroke, traumatic brain injury, rare diseases, neuroprotection). The alliance is also reflected through the ’Flying Faculty’ program developed by the WFNR with the support of EFNR. This initiative enables highly skilled experts to implement training programs in different countries, particularly regions where neurorehabilitation could be improved significantly.

## Leading Voices: Expert Perspectives on Neurorehabilitation

**Figure 2:**
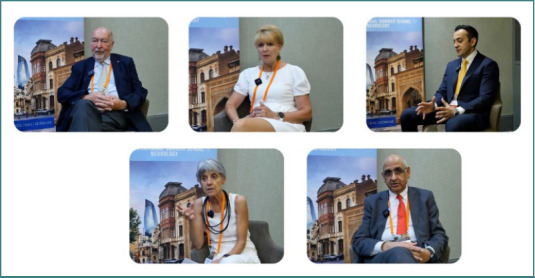
Interviews with leading experts were conducted during the event. From left to right - first row: Prof. Antonio Federico, Chairman of the Specialist Group on Rare Neurological Diseases within the World Federation of Neurology; Prof. Maria Judit Molnar, Professor of Neurology, Psychiatry, Clinical Genetics, and Clinico-pharmacology and director of the Semmelweis University’s Institute of Genomic Medicine and Rare Disorders in Hungary; Dr. Parvin Akbarov, advisor to the chairman and administration of TABIB and member of the board of directors at YANI Clinic; Prof. Paola Marangolo, Professor in Neuropsychology and Cognitive Neuroscience at the Department of Humanities Studies from the University Federico II in Naples; Prof. Sabahat Wasti, Medical Director and Staff Physician in Neurorehabilitation at the Cleveland Clinic Abu Dhabi in the United Arab Emirates.

Interviews with leading experts (Figure 2) revealed key advancements, challenges, and opportunities in rare neurological disorders, speech and language therapy, genomics, and stroke neurorehabilitation.

**Professor Antonio Federico**, the Chairman of the Specialist Group on Rare Neurological Diseases within the WFN, provided an overview of rare diseases, a “*Pandora's box for neurology and neuroscience*” that represents a great model for understanding brain function and dysfunction. Prof. Federico offered insight into his work within the WFN, pinpointing discrepancies among regions regarding access to diagnosis. He touched upon the activity at the research center at the University of Sienna, Italy, and highlighted central elements for access to care for patients with rare neurological diseases worldwide, discussing discrepancies in diagnosis and treatment, and underlining the roles of international collaboration and telemedicine.

**Prof**. **Maria Judit Molnar**, Professor of Neurology, Psychiatry, Clinical Genetics, and Clinico-pharmacology and director of the Semmelweis University’s Institute of Genomic Medicine and Rare Disorders in Hungary, shared her thoughts on the impact of technology in genomics and rare neurological diseases. She discussed the impact of artificial intelligence on future research and clinical practice pointing out that "*Artificial intelligence opened a new era for us, from prediction, prevention, through diagnosis, treatment, and follow-up*." Prof. Molnar elaborated on the national research center established in Hungary, emphasizing its potential for knowledge dexchange.

**Prof. Paola Marongolo**, Professor in Neuropsychology and Cognitive Neuroscience at the Department of Humanities Studies from the University Federico II in Naples, Italy, discussed the latest developments in speech and language therapy, including the roles of neurotechnologies in supporting recovery, and offered considerations on the importance of treatment intensity in aphasia. Prof. Marongolo pinpointed the importance of family and community-based approaches in treating aphasia, underscored the impact of evidence-based non-pharmacological interventions, and addressed the relevance of recognizing symptoms early on.

**Professor Sabahat Wasti**, Medical Director and Staff Physician in Neurorehabilitation at the Cleveland Clinic Abu Dhabi in the United Arab Emirates, discussed limitations in stroke rehabilitation on a global level and the importance of human resources and stroke centers. With regards to balance and gait disorders, Prof. Wasti addressed the need for careful assessment and personalized treatment in the context of a wide variety of symptoms as “*All interventions should be tailored to patients' requirements and needs*”. Lastly, he offered some considerations for overcoming limitations in neurorehabilitation.

During an exclusive interview, Dr. Parvin Akbarov, advisor to the chairman and administration of TABIB and member of the board of directors at YANI Clinica, emphasized the value of this event in Central Asia and its impact on global health management. He underscored the importance of networking opportunities, knowledge exchange, and building capacity in specialized fields such as neurorehabilitation, all of which contribute to improving the healthcare landscape. Furthermore, Dr. Akbarov discussed the pivotal role of TABIB in improving health services in Azerbaijan.

The interviews will be made available on EFNR’s YouTube and Blog.

All interviewed faculty addressed the added value of teaching courses in Central Asia and its capitalization at the local level. The experts highlighted the role of the course in improving awareness, supporting international collaboration, and facilitating research in rare diseases and neurorehabilitation.

## Advancing Regional Healthcare Perspectives: Administration of the Regional Medical Divisions in Azerbaijan

**Figure 3:**
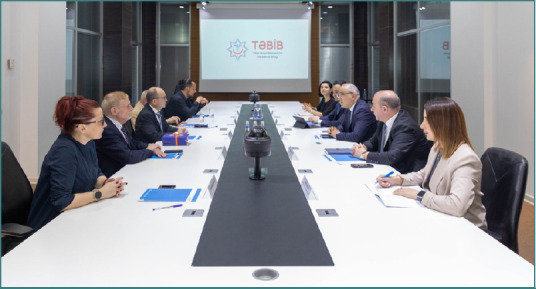
Meeting at the Administration of the Regional Medical Divisions in Azerbaijan (TABIB)

A TABIB meeting (Figure 3) brought together Prof. Mureșanu, Prof. Hömberg, Prof. Bornstein, Ms. Alexandra Gherman, AMN Executive Director, Mr. Cristian Andriescu, EFNR Executive Director, Mr. Araz Nasirov, TABIB Deputy Executive Director, Mr. Anar Aghayev, Deputy Director of the New Clinic and Head of the Somatics and STROKE Center, and Dr. Parvin Akbarov Advisor to the Executive Director. The guests were introduced to TABIB’s activities, including various initiatives undertaken, the implementation of compulsory health insurance, and innovations in service organization, alongside the principles guiding the management of medical institutions. The meeting also provided an overview of rehabilitation services, underscoring the institution’s commitment to enhancing healthcare delivery. Professor Hömberg highlighted WFNR’s aims and how these are achieved, focusing on the importance of developing and strengthening relationships among national and international institutions concerned with neurorehabilitation. On behalf of the EFNR, Prof. Dafin Mureșanu presented a model of cooperation, based on strategies elaborated between European scientific societies and regional entities. The discussions focused on comprehensive approaches to successful rehabilitation, from pre-hospital to long-term care, emphasizing the importance of multidisciplinary approaches. The meeting concluded with a mutual agreement to enhance cooperation at all levels by organizing joint events and supporting knowledge exchange among specialists in the field. Last but not least, within this model of cooperation mentioned by Prof. Mureșanu, the core element would be the creation of EFNR and AMN subsidiaries at national levels.

## Conclusions

Neurorehabilitation is becoming increasingly important as the burden of preventable neurological disorders continues to grow. From traditional clinical practice to emerging cutting-edge technologies, this dynamic field lies at the intersection of multiple disciplines and builds upon continuous advancements in education, research, and clinical practice.

Knowledge exchange and strenghtened collaboration among entities concerned with neurorehabilitation worldwide is essential for ensuring the best patient outcomes and enhancing quality of life. Only through collective efforts to address global challenges can the full potential of neurorehabilitation be achieved.
