# Supplementary material for: Neurorehabilitation Across Continents: the WFNR-EFNR Regional Meeting in conjunction with the 19th Congress of the Society for the Study of Neuroprotection and Neuroplasticity and the 19th International Summer School of Neurology in Baku, Azerbaijan
Source: J Med Life. 2024 Sep;17(9):825–9. doi: 10.25122/jml-2024-1014 (PMC11611057; doi:10.25122/jml-2024-1014)
Supplement: Supplementary file 1 [file JMedLife-17-825-s001.pdf]

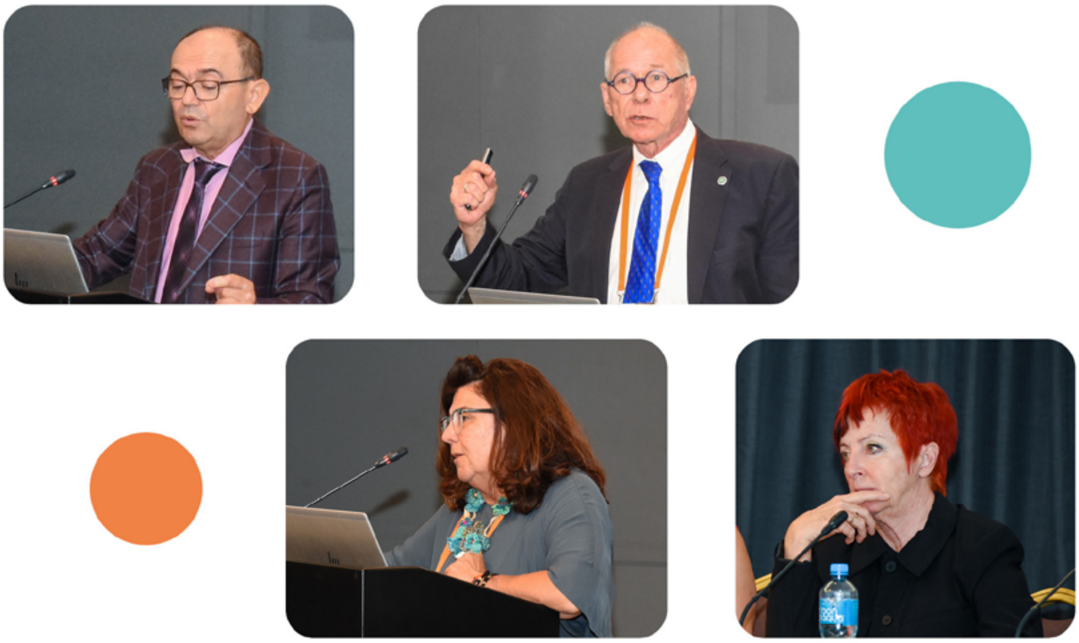

A. EFNR Core Presidium: Prof. Dafin Mureșanu, EFNR President, Prof. Volker Hömberg, EFNR Vice-president and WFNR President, Prof. Caterina Pistarini, Treasurer and Head of Research and Education Affairs, and Dr. Dana Boering, Secretary General

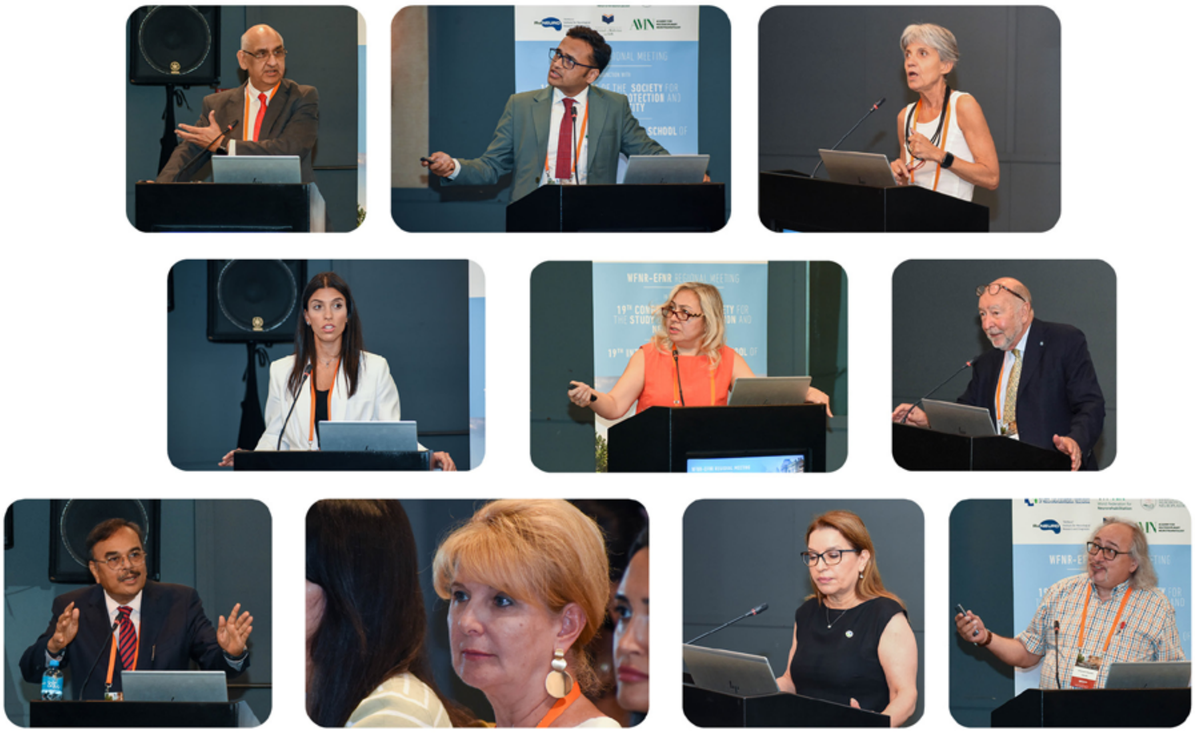

B. Speakers at the WFNR-EFNR Regional Meeting in conjunction with the 19th Congress of the Society for the Study of Neuroprotection and Neuroplasticity and the 19th International Summer School of Neurology. From left to right – first row: Prof. Sabahat Wasti, Medical Director and Staff Physician in Neurorehabilitation at the Cleveland Clinic Abu Dhabi in the United Arab Emirates, Dr. Vishal Pawar, Head of the Vertigo Clinic in Dubai, Prof. Paola Marangolo, Professor in Neuropsychology and Cognitive Neuroscience at the Department of Humanities Studies from the University Federico II in Italy; second row: Ms. Nour Salman, Vestibular and maxillofacial rehabilitation specialist at Cleveland Clinic Abu Dhabi, Prof. Nilda Turgut, Professor at Tekirdag Namik Kemal University in Turkey, Prof. Antonio Federico, Chairman of the Specialist Group on Rare Neurological Diseases within the World Federation of Neurology; bottom row: Professor Nirmal Surya, Chairman of Surya Neuro Centre and Founder Trustee and Chairman of Epilepsy Foundation, India, Prof. Maria Judit Molnar, Professor of Neurology, Psychiatry, Clinical Genetics, and Clinico-pharmacology and director of the Semmelweis University's Institute of Genomic Medicine and Rare Disorders in Hungary, Assoc. Professor Sadaquat Husseynova, Azerbaijan Medical University, and Prof. Slawomir Michalak, Head of the Stroke Unit at University Hospital in Poznan, Poland.

© 2024 by the authors. This article is an open access article distributed under the terms and conditions of the Creative Commons Attribution (CC BY 4.0) license.

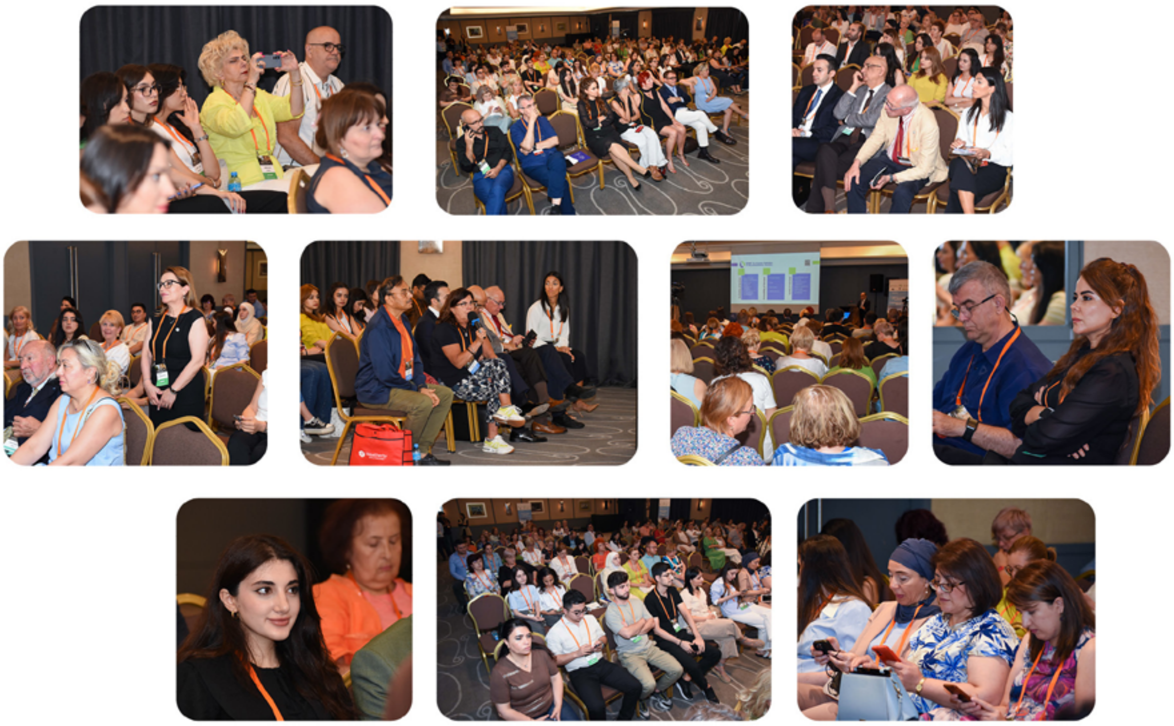

C. Participants and speakers at the WFNR-EFNR Regional Meeting in conjunction with the 19th Congress of the Society for the Study of Neuroprotection and Neuroplasticity and the 19th International Summer School of Neurology

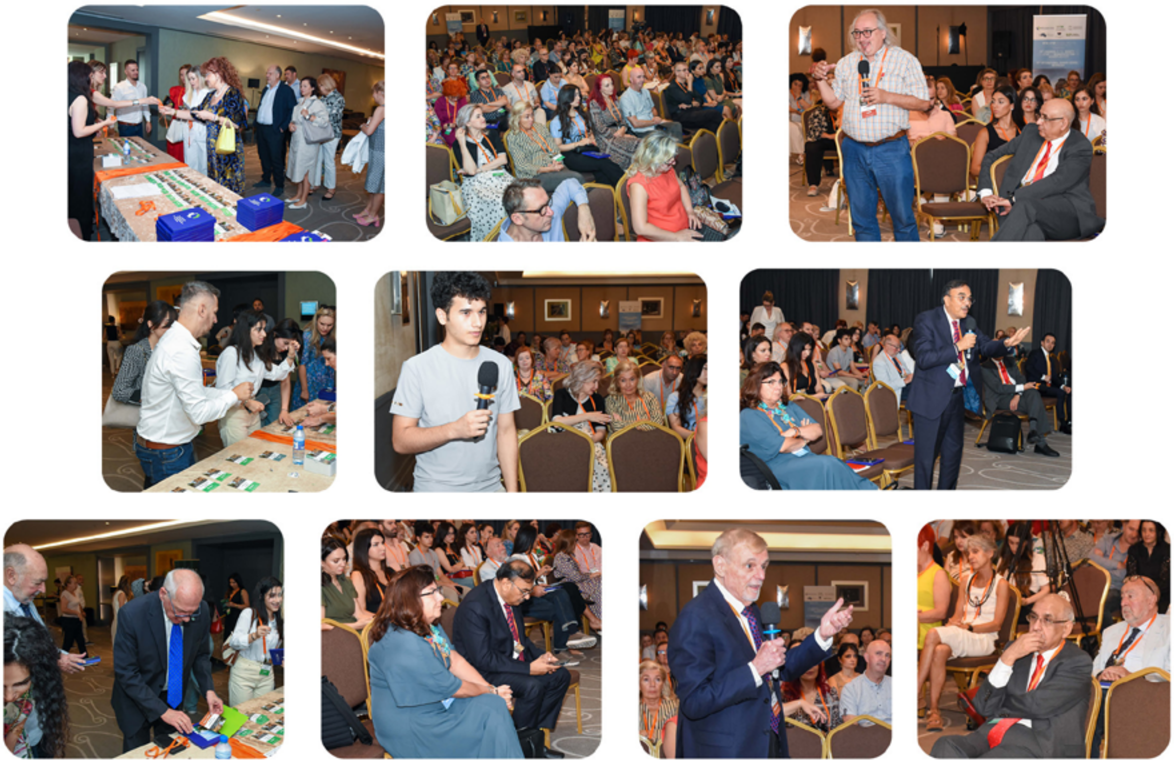

D. Faculty and participants at the WFNR-EFNR Regional Meeting in conjunction with the 19th Congress of the Society for the Study of Neuroprotection and Neuroplasticity and the 19th International Summer School of Neurology

© 2024 by the authors. This article is an open access article distributed under the terms and conditions of the Creative Commons Attribution (CC BY 4.0) license.

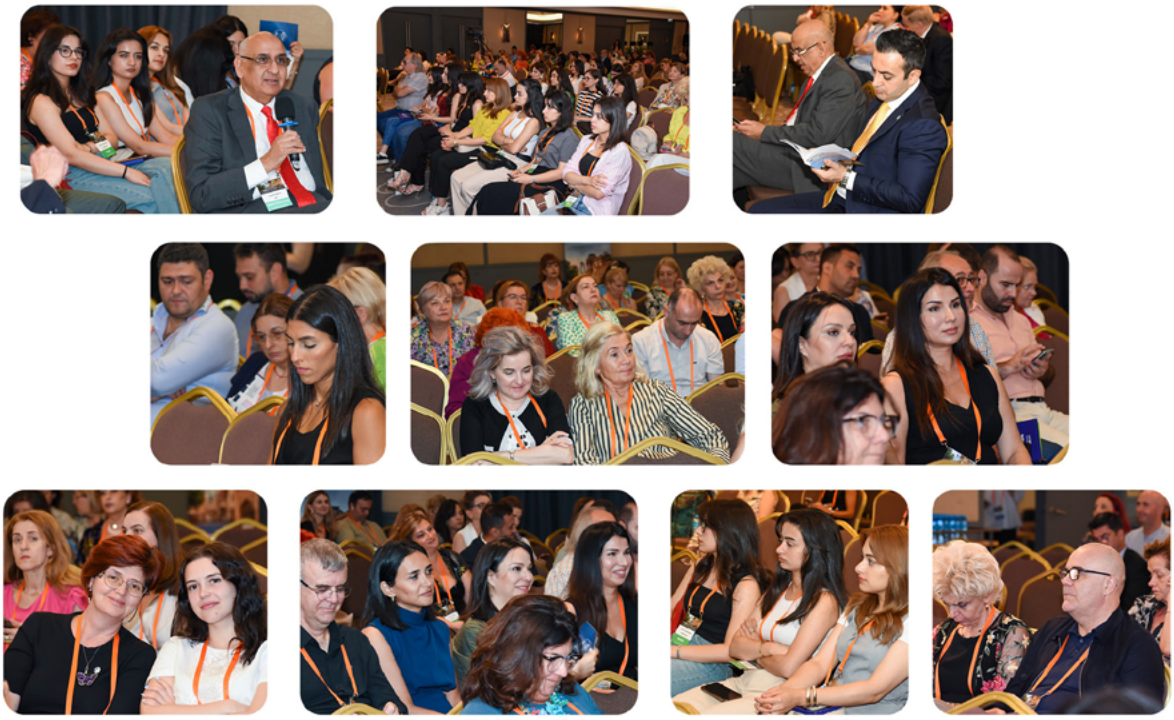

E. Speakers and participants at the WFNR-EFNR Regional Meeting in conjunction with the 19th Congress of the Society for the Study of Neuroprotection and Neuroplasticity and the 19th International Summer School of Neurology

© 2024 by the authors. This article is an open access article distributed under the terms and conditions of the Creative Commons Attribution (CC BY 4.0) license.
